# *Rhodiola rosea* Exerts Antiviral Activity in Athletes Following a Competitive Marathon Race

**DOI:** 10.3389/fnut.2015.00024

**Published:** 2015-07-31

**Authors:** Maryam Ahmed, Dru A. Henson, Matthew C. Sanderson, David C. Nieman, Jose M. Zubeldia, R. Andrew Shanely

**Affiliations:** ^1^Department of Biology, Appalachian State University, Boone, NC, USA; ^2^Human Performance Laboratory, Appalachian State University, Kannapolis, NC, USA; ^3^PoliNat SL, Las Palmas, Spain; ^4^Department of Health and Exercise Science, Appalachian State University, Boone, NC, USA

**Keywords:** *Rhodiola rosea*, physical activity, vesicular stomatitis virus, *Escherichia coli*, *Staphylococcus aureus*, bacterial growth

## Abstract

*Rhodiola rosea*, a medicinal plant with demonstrated adaptogenic properties, has recently been reported to contain active compounds with antimicrobial activity. The goal of this study was to measure the antiviral and antibacterial properties of the bioactive metabolites of *Rhodiola rosea* in the serum of experienced marathon runners following supplementation. Marathon runners, randomly divided into two groups, ingested 600 mg/day of *Rhodiola rosea* (*n* = 24, 6 female, 18 male) or placebo (*n* = 24, 7 females, 17 males) for 30 days prior to, the day of, and 7 days post-marathon. Blood serum samples were collected the day before, 15 min post-, and 1.5 h post-marathon. Serum from *Rhodiola rosea*-supplemented runners collected after marathon running did not attenuate the marathon-induced susceptibility of HeLa cells to killing by vesicular stomatitis virus. However, the use of *Rhodiola rosea* induced antiviral activity at early times post-infection by delaying an exercise-dependent increase in virus replication (*P* = 0.013 compared to placebo). Serum from both groups collected 15 min post-marathon significantly promoted the growth of *Escherichia coli* in culture as compared to serum collected the day before the marathon (*P* = 0.003, all subjects). Furthermore, the serum from subjects ingesting *Rhodiola rosea* did not display antibacterial properties at any time point as indicated by a lack of group differences immediately (*P* = 0.785) or 1.5 h (*P* = 0.633) post-marathon. These results indicate that bioactive compounds in the serum of subjects ingesting *Rhodiola rosea* may exert protective effects against virus replication following intense and prolonged exercise by inducing antiviral activity.

## Introduction

*Rhodiola rosea* (also known as golden root, rose root, Arctic root) is a perennial herbaceous plant in the Crassulaceae family found at high altitudes in the Arctic and mountainous regions throughout Europe and Asia (1). It has historically been used to treat a range of conditions, including fatigue, depression and anxiety, impotence, and nervous system disorders (1). *Rhodiola rosea* has also received attention for its potential adaptogenic and ergogenic properties including the enhancement of physical and mental performance and reduction of fatigue (2, 3). More recently, *Rhodiola rosea* and polyphenolic bioactive compounds from the plant, such as rosavin, salidroside, syringin, triandrin, and tyrosol, have been reported to exert beneficial effects against several pathogens. For example, flavonoids isolated from the roots of *Rhodiola rosea* have been shown to inhibit the activity of neuraminidases from *Clostridium perfringens* and influenza virus, and exert anti-influenza virus activities *in vitro* (4). Salidroside, a major bioactive component of *Rhodiola rosea* has been reported to modulate the expression of antiviral cytokines, such as IFNγ, and TNFα, and inhibit the replication of coxsackievirus B3 perhaps through modulation of oxidative stress (5, 6). More recently, studies have shown that *Rhodiola* inhibits dengue virus multiplication by regulating the innate immune response genes, RIG-I, MDA5, and ISG, to promote an effective antiviral immune response (7). Taken together, these data highlight the antiviral capacity of *Rhodiola rosea* and its potential as a therapeutic strategy against a variety of infectious diseases.

Although *in vitro* studies have yielded promising results, the antimicrobial capacity of *Rhodiola* species in humans has not been evaluated. The purpose of this study was to measure the antiviral and antibacterial activity in the serum of athletes ingesting *Rhodiola rosea* or placebo for 30 days prior to participating in a competitive marathon. This work is an extension of a study investigating the adaptogenic potential of *Rhodiola Rosea* in experienced runners completing a marathon (8). Shanely et al. reported that ingesting *Rhodiola Rosea* supplements for 30 days prior to marathon running did not reduce the muscle damage and inflammation generated by strenuous exercise. Intense endurance exercise, including marathon-type exertion, is known to induce deleterious changes in the immune system. During strenuous exercise, there is a substantial increase in reactive oxygen species (ROS) production resulting in oxidative stress, structural damage to the skeletal muscle fiber ultrastructure, inflammation, and transient immune dysfunction (9). Transient immunosuppression induced by prolonged, intense exercise is associated with increased incidence and severity of acute respiratory infections. Studies have observed decreased salivary IgA levels and natural killer (NK) cell function in endurance athletes, both of which offer protection against respiratory viruses, such as influenza virus and rhinoviruses (10–12). Therefore, there is great interest in determining efficacious countermeasures for athletes in order to reduce the risk of acquiring infections following strenuous exercise.

There are limitations to measuring the anti-pathogenic effects of therapeutics in human subjects due to ethical constraints. Because of this, we have developed *in vitro* assays to evaluate the ability of serum from supplemented individuals to inhibit the growth of bacteria, and the replication and cytopathic effects (CPE) associated with virus infections, *ex vivo*. Our previous studies using these techniques have yielded consistent and reproducible results allowing us to carry out human studies safely and non-invasively (13). In this study, we determined that *Rhodiola rosea* suppresses an exercise-induced increase in virus replication, but has no effect on the growth of the bacteria, *Escherichia coli* (*E. coli*) and *Staphylococcus aureus* (*S. aureus*). Consistent with previous reports, our data demonstrate that *Rhodiola rosea* contains compounds that act as antiviral agents.

## Materials and Methods

### Subjects

The details of the subjects and research design associated with this study have been previously published (8). The subjects participated in the Thunder Road Marathon on November 17, 2012 in Charlotte, NC, USA. Of the 55 volunteers, 48 runners completed all aspects of the study. Subjects were trained competitive runners with a marathon personal record of 4.5 h or less within the last 1.5 years, 25–65 years of age, and willing to avoid the use of additional supplements or medications known to affect inflammation. All study procedures were reviewed and approved by the Appalachian State University Institutional Review Board (IRB).

### Study design

*Rhodiola rosea* root extract was prepared by PoliNat SL (Las Palmas, Spain).*Rhodiola rosea* roots were obtained and prepared into powder by trained personnel in Siberia. The powder was transported to PoliNat manufacturing facilities in Las Palmas, Spain within 6 weeks. The bioactive content of the *Rhodiola rosea* powder was determined by high-performance liquid chromatography and extracted using a water:ethanol process. The final dried product was subjected to liquid chromatography for determination of the concentration of bioactive compounds. Results revealed a total rosavins content of >5% and salidroside >1.8%.

**Figure 1:**
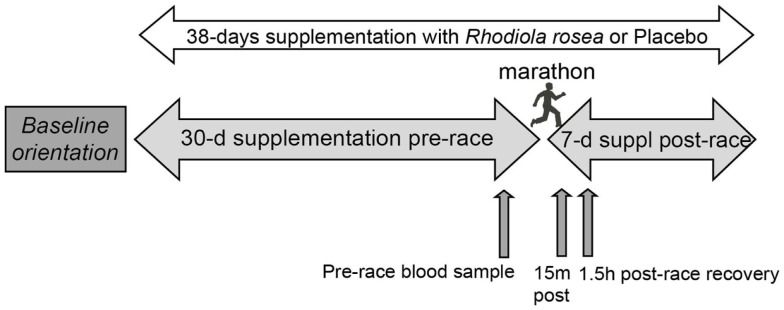
**Research design**. Randomized runners ingested *Rhodiola rosea* (*N* = 24) or placebo (*N* = 24) for 30 days prior to the marathon, the day of the marathon, and for 7 days after the marathon. Blood samples were collected the day before the marathon (pre-race), approximately 15 min post-marathon, and 1.5 h post-marathon. Serum collected from the blood was stored at −80°C until use.

Subjects received *Rhodiola rosea* extract or placebo capsules (prepared and supplied by PL Thomas & Co., Inc., Morristown, NJ, USA) in a randomized, double-blind study. Each *Rhodiola rosea* capsule contained 300 mg of *Rhodiola Rosea* extract containing 5.2% bioactives (11.3 mg of rosavins and 4.3 mg of salidroside). Each placebo capsule contained 300 mg cornstarch. Subjects ingested two capsules daily, one in the morning before breakfast and one in the evening before dinner for 30 days prior to the marathon, the day of the marathon, and for 7 days after the marathon. Blood samples were collected between 9:30 and 1:00 p.m. the day before the marathon, 2–15 min post-marathon, and 1.5 h post-marathon (Figure 1). Serum collected from the blood was aliquoted, snap frozen in liquid nitrogen, and stored at −80°C until use.

The *Rhodiola rosea* and placebo group did not differ significantly in age (40.3 ± 1.44 and 43.9 ± 1.23, respectively, *P* = 0.141). Additional subject characteristics, such as weight, BMI, body fat, and marathon time, also did not differ significantly between groups (8).

### Cells and virus

HeLa cervical cancer cells were cultured in Dulbecco’s modified Eagle’s medium (DMEM) containing 7.5% fetal bovine serum (FBS). Vesicular stomatitis virus (VSV) is a prototype of negative-strand RNA viruses, such as rabies and influenza viruses. It is highly cytopathic for cells from a variety of different species, including neurons and respiratory epithelial cells, thus making it a desirable model for the study of pathogenic viruses (14). Recombinant wild-type VSV (rwt virus) contains genes from several wt strains of VSV. Rwt virus and recombinant VSV expressing EGFP (green fluorescent protein) as a foreign gene (rwt-GFP virus) have been described previously (15). Virus stocks were prepared in HeLa cells using methods described previously (16). For all virus infections, HeLa cells were infected in DMEM with 7.5% FBS at a multiplicity of infection (MOI) of 3 PFU/cell.

### Antiviral activity

The ability of VSV to kill permissive cells in the presence of various concentrations of serum was measured in order to assess the antiviral properties in serum samples from *Rhodiola rosea* and placebo subjects. Specifically, serum samples at each time point were heat-inactivated for 30 min at 56°C (HI-serum) to prevent the complication of complement-mediated cell lysis. HeLa cells, susceptible to infection by VSV, were grown in 96-well dishes (in DMEM plus 7.5% FBS) until they reached approximately 70–80% confluency. At this time, cells were incubated with HI-serum at a serum to media ratio of 1:2. At various times post-incubation (0 and 4 h), cells were infected with rwt virus at a MOI of 3 PFU/cell. Cells were assessed for viability using MTT assay (MTT Cell Proliferation Kit, Roche Diagnostics) at 24 and 48 h post-infection. Controls included incubation of cells with serum alone (serum-alone samples) to measure potential cytotoxic effects of the serum, virus alone (virus-alone samples) to determine the cytotoxic effect of virus, and untreated and uninfected cells (mock) as background controls. Data were quantitated by dividing the values obtained from each sample by the mock value. All data were then normalized to the serum-alone data to adjust for differences in the serum responses between subjects. These values were then normalized to the pre-race values in order to more clearly observe changes following exercise and recovery periods.

### Virus replication

To determine the effect of serum from test subjects on the replication capacity of VSV, HeLa cells were grown in six-well dishes until they reached 70–80% confluency. As described above, cells were incubated with HI-serum at a serum to media ratio of 1:2. At 4 h post-incubation, cells were infected with rwt-GFP at an MOI of 3 PFU/cell. At 6 and 12 h post-infection, cells were washed, harvested in phosphate buffered saline (PBS), and subjected to flow cytometry to measure the fluorescence of GFP within cells. Two parameters were measured: (1) The percentage of cells expressing GFP, as an indication of the number of cells supporting virus replication, and (2) the geometric mean fluorescence, measuring the degree of replication within cells. All data were normalized to the pre-race values.

### Antibacterial activity

Serum samples from *Rhodiola rosea* and placebo supplemented subjects were tested for their effect on the growth of select bacteria. HI-serum samples were diluted at a 1:2 ratio with LB bacterial nutrient broth (Fisher Scientific) and incubated with 20 μl of overnight cultures of *Escherichia coli* (*E. coli*) strain K-12 or coagulase-positive *Staphylococcus aureus* (*S. aureus*), a BSL2 pathogen, both obtained from Carolina Biological Supply Company (Burlington, NC, USA). Controls included serum-alone samples, bacteria-alone samples (growth control), nutrient broth samples (sterility control), and bacteria grown in the presence of 128 μg/ml of ampicillin (obtained from Fischer Scientific). All experimental and control cultures were incubated at 37°C for 24 h, at which time samples were carefully re-suspended, transferred to a 96-well dish, and absorbance readings at 600 nm were measured (Molecular Devices SoftMax Pro 5 microplate reader). Data were quantitated by dividing the experimental samples (experimental minus serum-alone) by the growth control samples (growth control minus sterility control). As mentioned previously, these values were then normalized to the pre-race values.

### Statistical methods

Serum from male and female runners was combined for data analysis due to similarities in race time, muscle function, and inflammatory effects (8). All data are expressed as mean ± SE. Data were compared between groups at the two post-race time points using Student’s *t*-test, with comparisons between time points within a group compared using paired *t*-tests. After Bonferonni adjustment, statistical significance was set at *P* < 0.025, with trends noted at *P* < 0.05. Repeated measures ANOVA was not used since all data were normalized to pre-race values to adjust for differences in responses between subjects.

## Results

### Antiviral effects of *Rhodiola rosea* supplementation

**Figure 2:**
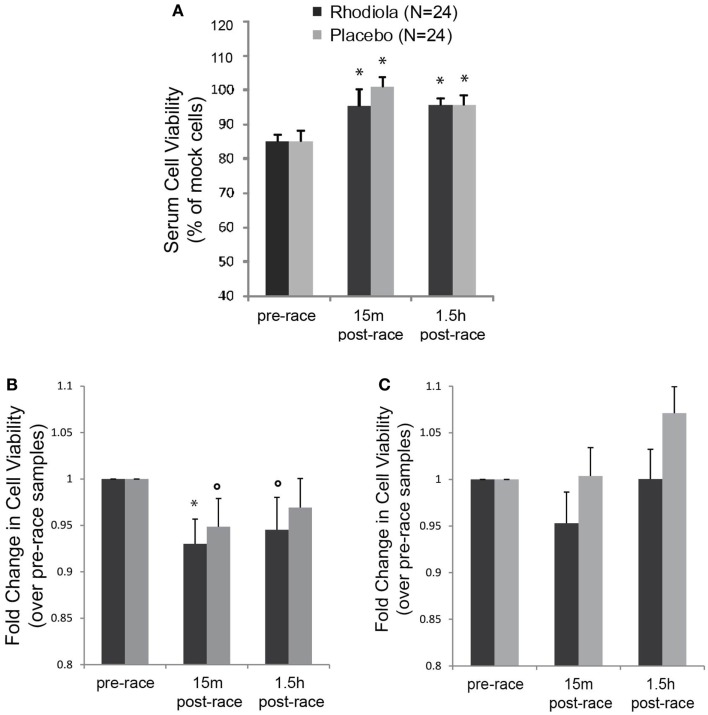
**Antiviral activity in the serum from test subjects**. HeLa cells were infected with serum from *Rhodiola rosea* or placebo- supplemented subjects for different times and infected with rwt virus. Cell viability was measured by MTT assay. Controls included cells infected with virus alone or incubated with serum alone. All data were normalized to mock-infected cells. **(A)** Impact of serum alone on viability of HeLa cells (24 h). **(B,C)**. Cells were incubated with serum from subjects for 0 h **(B)** or 4 h **(C)**, followed by VSV infection (rwt virus) for 24 h. Data were normalized to mock-infected cells and expressed relative to the pre-race values. Data are expressed as mean ± SE. *indicates significant differences relative to pre-race values (*P* < 0.025). ○indicates *P* < 0.05.

In order to measure the antiviral activity in serum from *Rhodiola rosea* and placebo supplemented individuals, we exposed HeLa cells to serum from marathon runners collected pre- and post-marathon and evaluated the ability of serum samples to protect cells against killing by VSV. Results show that there was no difference in the effect of serum alone from *Rhodiola rosea* versus placebo-supplemented subjects on HeLa cell viability at any time (Figure 2A). Furthermore, there was a significant increase in cell viability following exercise in both sample groups (15 min and 1.5 h post-race, *P* < 0.025). These data demonstrate that serum from subjects did not exert any cytotoxic effects on HeLa cells and that there were no group differences in cell viability. To measure the effect of serum on the viability of cells following virus infection, data were normalized to serum-alone samples and expressed relative to the pre-race values. Results in Figure 2B indicate that HeLa cells were more susceptible to killing by VSV when they were exposed to serum obtained from subjects after the marathon (5–7% decrease in cell viability, *P* < 0.05). This was apparent when cells were experimentally incubated with serum and virus at the same time (0 h). However, when cells were incubated with serum for 4 h prior to virus infection, we did not observe a decrease in HeLa cell viability (Figure 2C). In addition, serum from subjects who ingested *Rhodiola rosea* did not protect cells from the exercise-induced susceptibility to killing by rwt virus, as indicated by the lack of difference in cell viability between the two groups.

### Effect of *Rhodiola rosea* supplementation on virus replication

**Figure 3:**
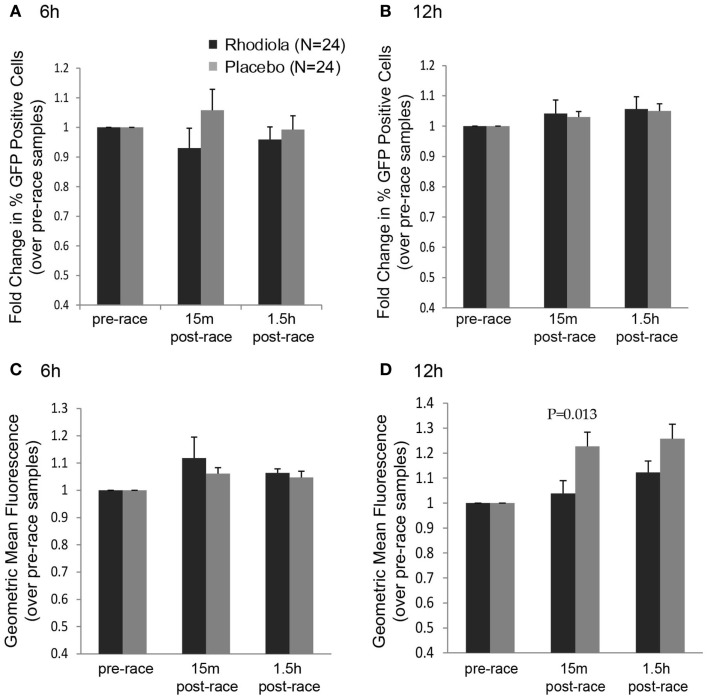
**Replication of VSV in HeLa cells in the presence of serum from *Rhodiola rosea* or placebo-supplemented subjects**. HeLa cells were incubated with serum from *Rhodiola rosea* or placebo subjects for 4 h and infected with rwt-GFP virus. At 6 or 12 h post-infection, GFP expression was measured by flow cytometry. Data were expressed as the percentage of cells expressing GFP at 6 h **(A)** or 12 h **(B)** and the geometric mean fluorescence at 6 h **(C)** and 12 h **(D)**. All results were normalized to mock-samples and expressed relative to the pre-race values. Data are expressed as mean ± SE. Data were compared between groups at each time point using Student’s *t*-test. *P* < 0.025 was considered statistically significant.

Although we did not observe any effect of supplementation on the ability of serum to protect cells from killing by VSV after completing a marathon, it is possible that the early effects of virus infection, such as steps in the viral replication cycle, were influenced by *Rhodiola rosea*. To measure the effects of *Rhodiola rosea*-supplemented serum on virus replication, HeLa cells were incubated with serum from subjects for 4 h prior to infection to allow cells to initiate cellular responses. Cells were then infected with an rwt virus strain engineered to express green fluorescent protein (rwt-GFP) during its replication cycle (15). Although not significant, a lower percentage of cells expressed GFP at 6 h post-infection when incubated with the 15 min post-race serum obtained from *Rhodiola rosea* versus placebo subjects (Figure 3A). This trend was not observed by 12 h post-infection (Figure 3B). Consistent with this result, at 12 h post-infection, the geometric mean fluorescence was significantly lower in cells exposed to the 15 min post-race serum from *Rhodiola rosea*-supplemented individuals as compared to serum from placebo subjects (*P* = 0.013) (Figure 3D). This difference was not observed at 6 h post-infection (Figure 3C). These results indicate that *Rhodiola rosea* delayed virus infection immediately after the marathon. Although virus recovered its ability to infect cells in culture by 12 h post-infection (Figure 3B), the degree of replication within cells was significantly lower during that time, as indicated by the geometric mean fluorescence data in Figure 3D.

### Effect of *Rhodiola rosea* on growth of bacteria

**Figure 4:**
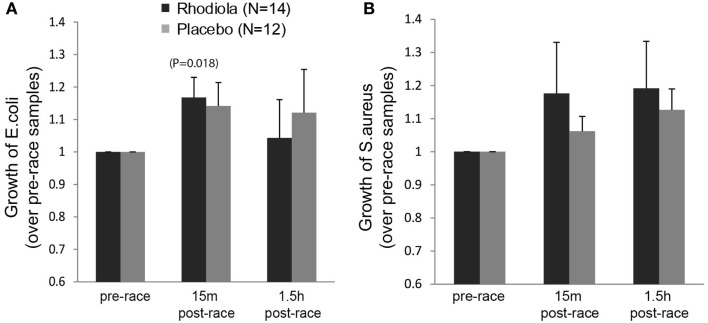
**Growth of bacteria in the presence of serum from *Rhodiola rosea* versus placebo-supplemented subjects**. *E. coli* **(A)** or *S. aureus* **(B)** was incubated with *Rhodiola rosea* or placebo serum for 24 h. Growth of bacteria was determined by measuring absorbance readings at 600 nm. All readings were normalized to the pre-race supplementation values. Data are expressed as mean ± SE. Time-points within a group were compared using paired *t*-test. *P* < 0.025 was considered statistically significant.

To determine the antibacterial effects of *Rhodiola rosea*, the effect of serum on the growth of the pathogenic bacteria, *E. coli* and *S. aureus*, was evaluated. Our results in Figure 4A show a significant exercise-induced increase in bacterial growth of *E. coli* in the *Rhodiola rosea* (16.8% increase, *P* = 0.018) group, as well as an increasing trend in the placebo (14.2% increase, *P* = 0.052) group, which decreased during the recovery period. Additionally, there were no significant group differences in the growth of *E. coli* (Figure 4A) or *S. aureus* (Figure 4B) 15 min or 1.5 h post-marathon. These data indicate that intense and prolonged exercise leads to increased susceptibility to bacterial infection, which is not improved by ingesting *Rhodiola rosea*.

## Discussion

Our results indicate that *Rhodiola rosea* supplementation has the potential to protect athletes from exercise-induced susceptibility to infections by attenuating virus replication. Studies have shown that an aqueous extract derived from the rhizome of *Rhodiola imbricata* induces the expression of the innate immune response genes, RIG-I and MDA5, in response to infection with dengue virus (7). RIG-I and MDA5 are cytoplasmic DEx (D/H) box helicases that function as pattern recognition receptors for the detection of intracellular viral products, such as viral nucleic acids, leading to the expression of type I interferons (IFN-α/β) (17, 18). Type I IFNs secreted from infected cells bind to the IFNα receptor (IFNAR) on surrounding cells to initiate transcription of IFN-stimulated genes with antiviral functions. Two key components of IFN-mediated action against viruses are 2′,5′-oligoadenylate synthetase (2′,5′-OAS) and MxA, which inhibit viral replication by inducing viral RNA degradation and inhibiting spread (19). RIG-I responds to a variety of positive and negative strand RNA viruses, including VSV (20). Therefore, it is possible that *Rhodiola rosea* regulates the antiviral response in infected cells in a manner similar to *Rhodiola imbricata* and this effect may be responsible for the decrease in virus replication that we observed when cells were incubated with serum from *Rhodiola rosea* versus placebo subjects. Future studies will investigate the effect of *Rhodiola rosea* extracts and supplementation on type I IFN induction by innate immune receptors and downstream effects on IFN signaling pathways.

We did not observe a significant effect of *Rhodiola rosea* on the ability of cells to delay the CPE associated with VSV infection *in vitro*. In our study, cells were infected synchronously with a wild-type (wt) strain of VSV expressing the viral matrix (M) protein. M protein inhibits host gene expression in infected cells resulting in the suppression of the cellular antiviral immune response (20). Therefore, *Rhodiola rosea* supplementation may have successfully delayed the early steps of the replication cycle of VSV in infected HeLa cells by inducing expression of antiviral factors, but the VSV M protein may have ultimately suppressed the beneficial effect of *Rhodiola rosea* by shutting off expression of antiviral genes. In support of this hypothesis, studies have reported that the inhibition of host gene expression by M protein in HeLa cells leads to cellular damage and stress resulting in the acceleration of cell death via the mitochondrial (intrinsic) apoptotic pathway (21, 22). It is possible that exposure of cells to a lower concentration of virus in our study would allow serum factors from *Rhodiola rosea*-supplemented subjects to have the opportunity to induce a more robust antiviral response and attenuate the replication and spread of VSV. Furthermore, *Rhodiola rosea* may exert more beneficial effects during natural viral infections, where cells would be exposed to lower levels of virus particles than those artificially administered in our cell culture study.

Limited studies have investigated the effect of *Rhodiola rosea* and extracts from *Rhodiola rosea* on infections by bacterial pathogens. Cybulska et al. reported that *Rhodiola rosea* extracts containing salidroside and rosavin demonstrated some inhibitory activity against *Neisseria Ghonorrhea* isolates with different antimicrobial resistance phenotypes (23). Although this study highlights a potential inhibitory effect of *Rhodiola* species on bacterial infections, in our study, serum from subjects ingesting *Rhodiola rosea*-collected post-marathon failed to suppress the growth of both *E. coli* and *S. aureus* in culture. In fact, marathon running stimulated the growth of *E. coli* in both placebo and *Rhodiola rosea* groups. These results are similar to our recent findings in which serum from athletes ingesting a blueberry–green tea–polyphenol soy protein complex (PSPC) promoted the growth of both *E. coli* and *S. aureus* similarly to the placebo group following a 3-day intensified training period (13). Interestingly, polyphenolic tea catechins are effective in decreasing expression of bacterial virulence factors, such as those responsible for biofilm formation and motility (24). Therefore, it is possible that salidroside and other phenolic compounds from *Rhodiola rosea*-supplemented subjects exert antibacterial effects that were not measured in our study, such as biofilm formation or changes at the level of gene expression. In support of this hypothesis, salidroside from *Rhodiola crenulata* was shown to be an active compound responsible for preventing *Propionibacterium acnes* biofilm formation *in vitro* (25). Additionally, since we did not measure the levels of polyphenols in the circulation at any time during our study, it is possible that levels of the active compounds in *Rhodiola rosea* may be below the minimum inhibitor concentration (MIC) for the bacteria tested in our study. Because studies have indicated that *Rhodiola* induces cellular changes that are antiviral in nature, it is likely that viruses and other intracellular pathogens may be more susceptible to the effects of *Rhodiola rosea* supplementation than extracellular pathogens. From a practical viewpoint, endurance athletes are prone to acute viral illnesses following heavy exertion, making the antiviral effects of *Rhodiola rosea* more applicable to the athletic condition.

During strenuous exercise, there is a substantial increase in ROS, which is not only associated with cell and tissue destruction, but with the regulation of immune responses at the level of both T cells and antigen-presenting cells (26). Oxidative stress and excessive production of superoxides has also been found to enhance infections associated with a variety of different viruses and promote tissue damage, perhaps by activation of the transcription factor NF-kβ leading to enhanced inflammation and apoptosis of infected cells (27). Therefore, naturally occurring nutritional sources of antioxidants containing polyphenolic compounds have the potential to an attenuate oxidative damage by acting as ROS scavengers, metal chelators, and enzyme modulators (26, 28). It is intriguing to consider that polyphenols in *Rhodiola rosea* may decrease the risk of acquiring virus infections following rigorous exercise by inhibiting oxidative stress. However, Shanely et al. showed that supplementation of our subject group with *Rhodiola rosea* did not lead to changes in the expression of extracellular heat shock protein (HSP) 72, a marker of oxidative stress, or the levels of inflammatory cytokines following marathon running (8). Therefore, it is possible that alternative mechanisms, such as those at the cellular level, may be responsible for the attenuation of virus replication that we observed in this study. Our future studies will be aimed at determining the molecular mechanisms by which *Rhodiola rosea* induces antiviral activity in athletes.

## Conflict of Interest Statement

The authors declare that the research was conducted in the absence of any commercial or financial relationships that could be construed as a potential conflict of interest.
